# Metabolomic Analysis Reveals Changes of Bioactive Compounds in Mung Beans (*Vigna radiata*) during γ-Aminobutyric Acid Enrichment Treatment

**DOI:** 10.3390/foods11101423

**Published:** 2022-05-13

**Authors:** Yuling Ma, Sumei Zhou, Jing Lu

**Affiliations:** 1 Beijing Advanced Innovation Center for Food Nutrition and Human Health, Beijing Key Laboratory of Flavor Chemistry, Beijing Technology and Business University, 11 Fucheng Road, Beijing 100048, China; yuling.ma@student.uliege.be (Y.M.); zhousumei@btbu.edu.cn (S.Z.); 2 School of Food Science, Henan Institute of Science and Technology, Xinxiang 453003, China; 3 Department of Food Science and Formulation, Gembloux Agro-Bio Tech, Université de Liège, Passage des Déportés 2, 5030 Gembloux, Belgium

**Keywords:** heat and relative humidity, GABA-enrichment processing, flavonoids, phenolic acids, α-ketoglutaric acid

## Abstract

Soaking together with Heat and Relative Humidity (HRH) treatment has been applied successfully to enrich γ-aminobutyric acid (GABA) in mung beans. However, whether and how the above GABA enrichment processing influences the other bioactive molecules is elusive. In the present study, mung beans were soaked and then treated by HRH for 5 or 7 h. By using metabolomics techniques, the changes of 496 metabolites were determined. The relative content of flavonoids and phenolic acids increased during soaking but slightly decreased during HRH. Intriguingly, soaking and HRH had the opposite effects on the glycosylation of polyphenols. The relative content of glycosylated or un-glycosylated polyphenols increased during soaking or HRH, respectively. The relative content of α-ketoglutaric acid increased more than 20 times after 5 h HRH treatment. Bioactive molecules could be enriched during GABA enrichment processing. Depending on the desired bioactive compounds, soaking and different duration of HRH treatment could be selected.

## 1. Introduction

Mung beans (*Vigna radiata*), a short-duration crop, are well-consumed legumes in Asia. They have been utilized for more than 3500 years all over the world [1]. Mung beans can be adapted to grow under various conditions of soil, including arid and semi-arid ones. They are rich in protein and dietary fiber while low in lipids. Mung bean proteins are easier to be digested compared to those of other legumes [2]. In addition, mung beans are rich in bioactive compounds, including phenolic acids, flavonoids, and polysaccharides, etc. [3,4]. In the traditional Chinese Compendium of Materia Medica, mung beans are well known to expel pathogenic factors and clear heat in summer [4]. Moreover, they have multiple potential health benefits, including hypoglycemic and hypolipidemic effects, antihypertensive function, hepatoprotection, neuroprotective activity, and immunomodulatory properties [5,6,7,8,9,10].

γ-aminobutyric acid, also known as GABA, is a non-protein synthesis amino acid. It is widely found in microorganisms, plants, animals, and humans [11]. Numerous food products, such as fruits and vegetables, also contain GABA. However, its content is typically too low for industrial use [11]. GABA is a well-known neurotransmitter inhibitor in the central neural system in humans and animals [12]. It also has other physiological functions, including anti-stress, antihypertension, antidiabetics, skin beauty care, anti-depression, and neuroprotective effect, etc. [13,14,15,16,17,18]. Thus, various treatments have been applied to increase the content of GABA in food. For example, germination and fermentation were frequently used to enrich GABA in grains [11]. However, normally these treatments are time-consuming and labor-intensive [19]. To overcome these drawbacks, Heat and Relative Humidity (HRH) treatment was established and applied in various food materials [20]. The content of GABA in black mung beans was upregulated 48 times after HRH treatments in our previous work [19]. However, besides the accumulation of GABA, the influences of HRH processing on the other bioactive compounds were still unknown. Previous reports suggested that flavonoids, phenolic acids, and organic acids contributed significantly to the antioxidative activity of mung beans [10,21]. It is thus worthy of investigating the changes of these compounds during GABA enrichment.

**Figure 1:**
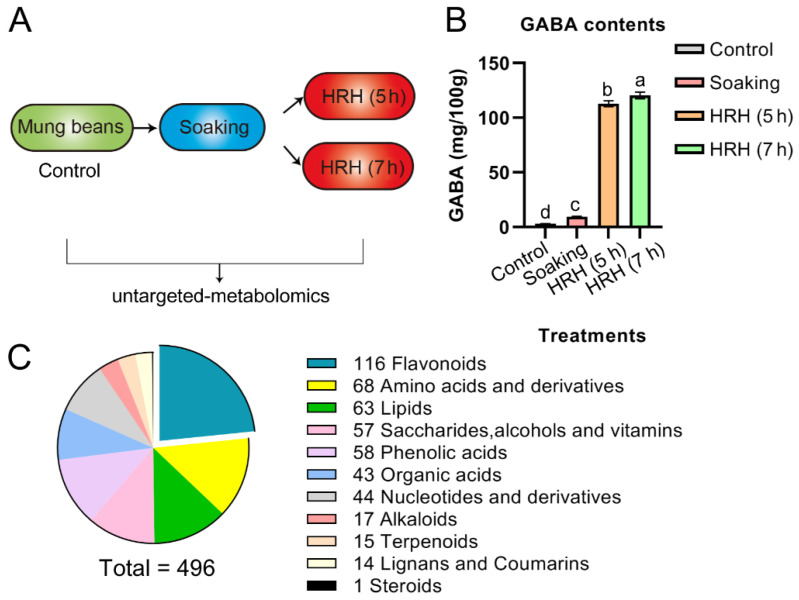
(**A**) Schematics depicting how to prepare the samples in this study. (**B**) GABA content during processing, a,b,c,d, *p* < 0.05. (**C**) Eleven types of metabolites were identified in mung beans during GABA-enrichment processing.

In the present study, soaking, short, and long time HRH treatment were applied for GABA accumulation (Figure 1A). The changes of 496 bioactive molecules during the treatments were analyzed by using a widely targeted metabolomics technique. This technique was firstly reported by Sawada et al. in 2009 [22]. Normally, the optimal ionization condition and fragment characteristics of each interested compound were determined. Furthermore, a local database with this information was established. Then, multiple reaction monitoring (MRM) mode was used to analyze the materials using high-throughput UPLC-MS/MS. Compared with targeted and non-targeted metabolomics, this technique is qualitatively and quantitatively accurate with high throughput, and high sensitivity [22]. The present study will deepen our understanding regarding the change of functional molecules during processing. The results will aid in selecting optimal procedures to produce mung bean foods with desired bioactive molecules and functions.

## 2. Materials and Methods

### 2.1. Materials

Black mung beans (Yuheilv No.3) were harvested in 2019 and obtained from Sandou Food Co., Ltd., Gucheng, China.

### 2.2. GABA-Enrichment Processing

According to the method of Fukumori et al. [20] and the results of our previous work [19], 50 g of black mung beans were first washed and then soaked in a sealed vessel at room temperature to make the moisture content of 25%. Then, black mung beans were treated at 65 °C, 98% relative humidity for 5 or 7 h, respectively (KW-TH-49T, Dongguan KOWIN Testing Equipment Co., Ltd., Dongguan, China). All the samples were immediately put into liquid nitrogen and stored at −80 °C for further analysis. All treatments were conducted in triplicates.

### 2.3. GABA Content Analysis

GABA content was determined according to the method of Yang et al. [23] with some modifications. Briefly, 70% ethanol was added to mung bean powder at room temperature and shook for 30 min. The supernatant layer was centrifuged at 11,000× *g*, 25 °C for 10 min. Then, 1 mL of the extraction was mixed with 0.2 mL NaHCO_3_ (0.4 g in 10 mL water) and 0.4 mL dabsyl chloride (20 mg in 10 mL acetonitrile). The mixture was incubated at 70 °C for 20 min. The GABA content was analyzed using HPLC (Agilent 1260, Agilent Technologies Inc., Santa Clara, CA, USA).

### 2.4. Widely-Targeted Metabolomics Techniques

#### 2.4.1. Sample Preparation and Extraction

Control, soaked, 5 h-HRH-treated and 7 h-HRH-treated freeze-dried mung beans were crushed at 30 Hz for 1.5 min using a mixing mill (MM 400, Retsch, Düsseldorf, Germany) with zirconia beads. Metabolites were then extracted from 100 mg mung bean powder with 0.6 mL of 70% methanol in water overnight at 4 °C. After centrifugation at 10,000× *g* for 10 min, the extract was absorbed (CNWBOND Carbon-GCB SPE Cartridge, 250 mg, 3 mL; ANPEL, Shanghai, China) and filtered (SCAA-104, 0.22 μm pore size; ANPEL, Shanghai, China). Extracts of all samples were pooled to prepare the mixed sample.

#### 2.4.2. Qualitative and Quantitative Analysis of Metabolites

Qualitative Analysis: the mixed sample was used for qualitative analysis by UPLC-MS/MS (TripleTOF 6600, AB SCIEX, Framingham, MA, USA). Agilent SB-C18 (1.8 µm, 2.1 mm × 100 mm) was used for UPLC. Gradient elution was applied and started with 95% A (0.1% formic acid in Milli-Q water) and 5% B (0.1% formic acid in acetonitrile). Within 9 min, the content of A in the mobile phase decreased to 5% and was kept for 1 min. Then, the elution solution was adjusted to 95% A within 1.1 min and kept for 14 min. The temperature of the column oven was 40 °C.

The data acquisition was operated using the information-dependent acquisition (IDA) mode (TripleTOF 6600, AB SCIEX, Framingham, MA, USA). The source parameters were set as follows: ion source gas 1, 50 psi; ion source gas 2, 50 psi; curtain gas, 25 psi; temperature, 500 °C; de-clustering potential, 60 V, or −60 V in positive or negative modes, respectively; and ion spray voltage floating, 5500 or−4500 V in positive or negative modes, respectively. The TOF MS scan parameters were set as follows: mass range, 50–1000 Da; accumulation time, 200 ms. The product ion scan parameters were set as follows: mass range, 50–1000 Da; accumulation time, 50 ms; collision energy, 30 or −30 V in positive or negative modes, respectively, mass tolerance, 50 ppm.

The identification of metabolites was based on the accurate mass, MS^2^ fragments, MS^2^ fragment isotope distribution and retention time (RT). The MS, MS^2^ spectrum and RT of metabolites were compared to home-built database (MWDB, Wuhan Metware Biotechnology Co., Ltd., Wuhan, China) and the MS tolerance and MS^2^ tolerance were set to 20 ppm.

Quantitative analysis: all samples were analyzed by UPLC-MS/MS (AB SCIEX 4500 QTRAP, Framingham, MA, USA), and the mixed sample was used to check the stability of the instrument and injected after every 3 injections. The setting of UPLC was the same as that of qualitative analysis. The metabolites were quantified by MRM mode of triple-quadrupole mass spectrometry (4500 QTRAP, Framingham, MA, USA). The ESI source operation parameters were set as follows: temperature 550 °C; ion spray voltage 5500 V (positive ion mode)/−4500 V (negative ion mode); ion source gas I, gas II, curtain gas was set at 50, 60, and 30.0 psi, respectively. The de-clustering potential and collision energy were optimized for each metabolite in MRM mode [24]. The peak area was integrated and manually checked for each substance. The peak area of metabolites was compared in different samples.

### 2.5. Statistical Analysis

One-way Anova (Fisher’s Least Significant difference) was applied to compare the differences among groups (PASW statistics 18, Armonk, NY, USA). A hieratical cluster was used to investigate the change of individual compounds during processing. The data were preprocessed using k-means before applying clustering and generating a heatmap. Euclidean distance and average linkage were selected for clustering process (Perseus 1.5.0.15).

## 3. Results

### 3.1. The Amount of GABA during Processing

As exemplified in Figure 1B and Table 1, the amount of GABA increased to 3.3 times during soaking and further increased to 39 and 42 times after HRH treatment for 5 h and 7 h, respectively. The above results suggested that soaking in combination with HRH treatment was sufficient to enrich GABA in mung beans.

### 3.2. Identified Metabolites and Their Changes during GABA-Enrichment Processing

In total, 496 metabolites (Supplementary Table S1), which can be categorized into 11 subtypes, were identified and quantified during GABA enrichment processing (Figure 1C). Among identified subtypes, flavonoids accounted for the largest portion, followed by amino acids, lipids, saccharides, alcohols and vitamins, phenolic acids, and organic acids, etc. (Figure 1C). The total peak area of metabolites of the 11 subtypes was compared during GABA enrichment (Table 1). The trends of the above 11 subtypes were as follows: (1), the relative concentration of total flavonoids significantly increased after soaking compared with pre-soaking beans (i.e., control group), and gradually decreased as the time of HRH treatment increased. However, it was still significantly higher than that of the control group. We further divided flavonoids into seven different subgroups (i.e., flavonols, flavones, isoflavones, flavanones, flavanols, chalcones, and proanthocyanidins in Table 1). The relative concentration of flavonols was highest after soaking and significantly decreased to lower than that of the control after 7 h HRH treatment. The relative content of flavones increased after soaking and decreased after HRH treatment. However, it was still higher than that of the control. The relative content of isoflavones reached the highest level after soaking and decreased to a similar level of control after HRH treatment. For flavanones, their relative content rose during soaking and remained the same during HRH treatment. (2) The relative content of free amino acids and their derivatives significantly increased during soaking and reached the highest level after 7 h-HRH treatment. The same trend was observed for the subgroup- essential amino acids. (3) The relative concentration of total organic acids in the soaked beans increased 1.35 times and decreased to a similar level to the control group after HRH treatment. (4) The relative concentration of terpenoids significantly increased during soaking and decreased to lower than that of the control group after HRH treatment. (5) Total lipids decreased significantly after soaking, but HRH treatment restored it to a similar level to the control group. The same trend was observed for total free fatty acids. The relative content of phospholipid was higher in the control and HRH-treated mung beans. (6) The relative level of total saccharides, alcohols, and vitamins did not change during soaking but significantly increased after HRH treatment. (7) The relative concentration of alkaloids only significantly increased after 7 h HRH treatment. (8) Moreover, total nucleotides and their derivatives, lignans, and cholesterols did not change during processing.

### 3.3. The Changes of Individual Metabolites during Processing

Fold of change of >1.5 times and *p*-value of < 0.05 were used as cutoffs to analyze differential metabolites among groups. Compared to the control group, soaking, short time HRH (5 h), and longtime HRH (7 h) significantly increased the relative concentration of 99, 137, and 171 metabolites, respectively, and significantly decreased the relative concentration of 19, 16, and 42 metabolites, respectively. In total, 271 compounds were significantly changed during processing, including 67 flavonoids, 37 amino acids and derivatives, 33 lipids, 20 saccharides, alcohols, and vitamins, 31 phenolic acids, 21 organic acids, 36 nucleotides and derivatives, 9 alkaloids, 8 terpenoids and 9 lignans, and coumarins.

#### 3.3.1. The Changes of Individual Flavonoids during GABA Enrichment Processing

**Figure 2:**
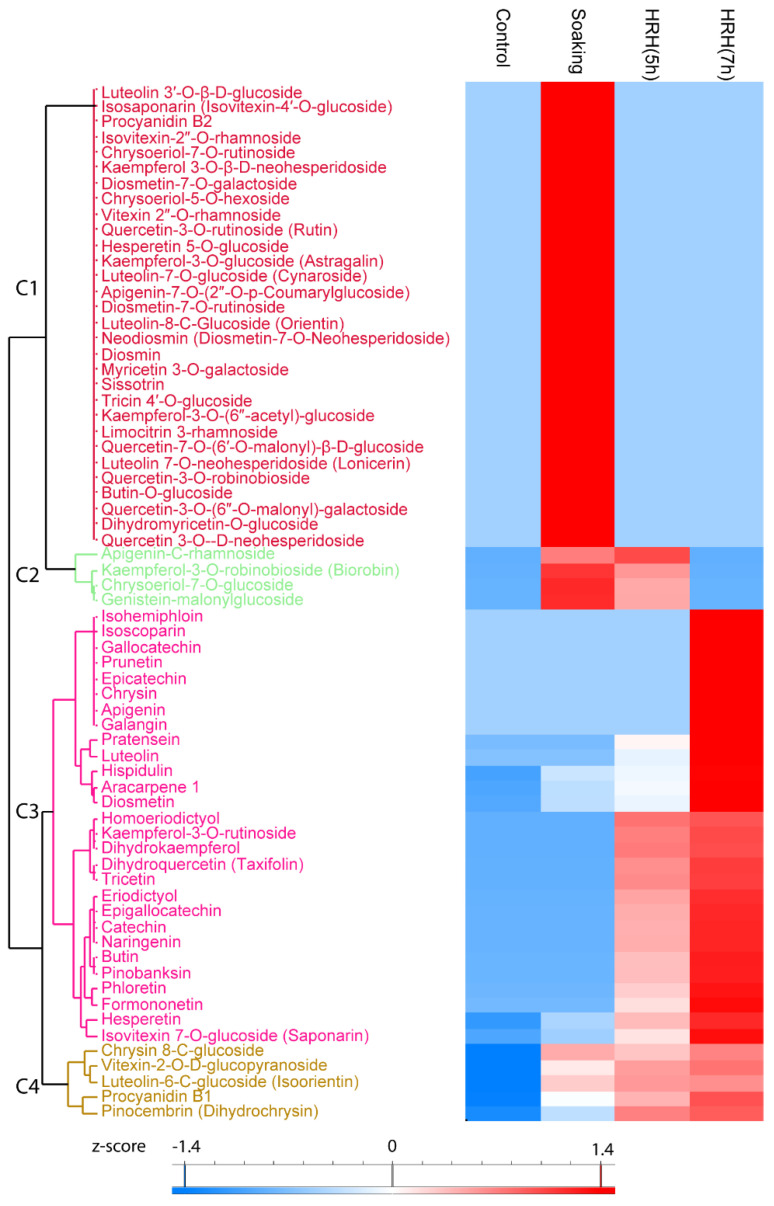
Hierarchical cluster analysis based on peak area of flavonoids. C1–C4: Cluster 1–4.

Sixty-seven flavonoids significantly changed during processing (Figure 2), 30 of them increased significantly during soaking (Figure 2, Cluster 1). Frequently reported mung bean flavonoids vitexin 2″-*O*-rhamnoside and quecetin-3-*O*-rutinoside were in this cluster. Moreover, four flavonoids, such as kaempferol-3-*O*-robinobioside and apigenin-*C*-rhamnoside, etc. (Figure 2, Cluster 2), significantly increased during soaking and short time HRH treatment. The relative concentration of 28 flavonoids was higher after HRH treatment (Figure 2, Cluster 3). Among them, the relative concentration of isohemiphloin, isoscoparin, gallocatechin, prunetin, epicatechin, chrysin, apigenin, and galangin significantly increased only after a long time HRH treatment. The level of pratensein, luteolin, homoeriodictyol, kaempferol-3-*O*-rutinoside, dihydrokaempferol, dihydroquercetin, tricetin, eriodictyol, epigallocatechin, catechin, naringenin, butin, pinobanksin, phloretin, and formononetin significantly increased after both short and long time HRH treatment. The relative content of hesperetin and isovitexin 7-*O*-glucoside increased after soaking, HRH (5 h) treatment and reached to the highest level after 7 h HRH treatment. In Cluster 4, the relative concentration of these flavonoids significantly increased after soaking and kept unchanged during HRH treatment, including one of the well-known flavonoids in mung bean iso-orientin (Figure 2, Cluster 4). Of note, the most abundant flavonoids reported in mung beans, vitexin, and isovitexin, were not changed during GABA enrichment.

#### 3.3.2. The Change of Amino Acids and Their Derivatives during GABA-Enrichment Processing

**Figure 3:**
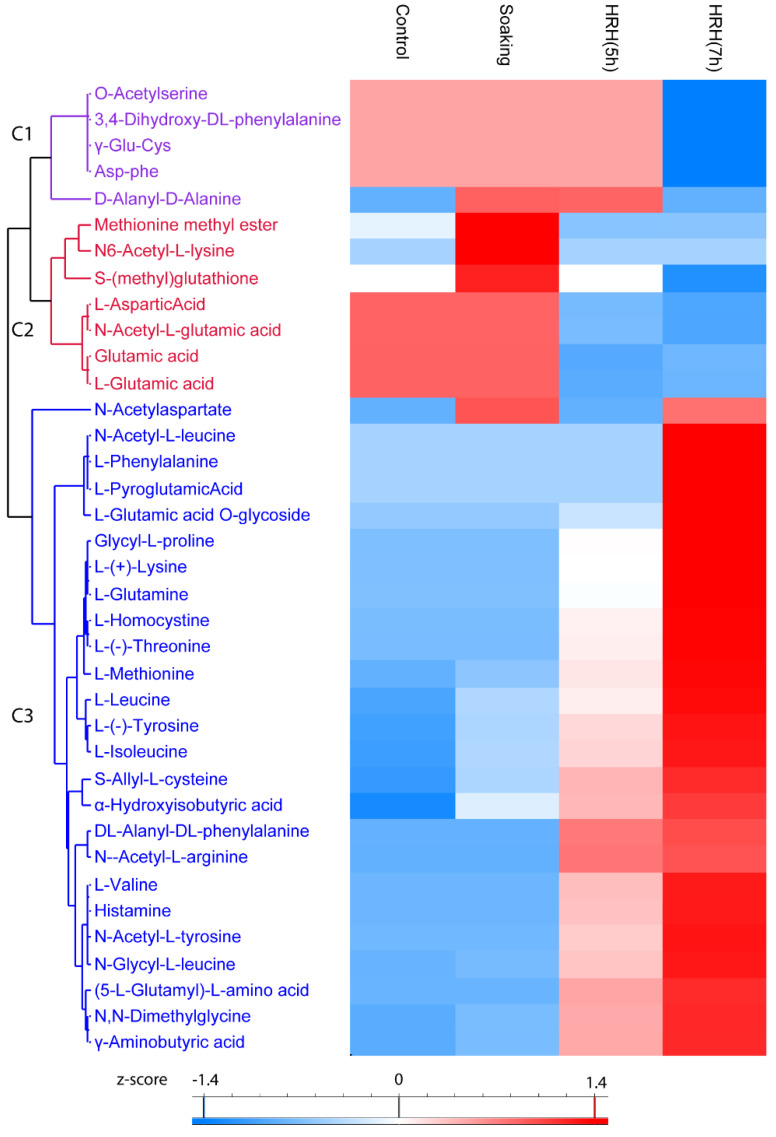
Hierarchical cluster analysis based on peak area of amino acids and its derivatives. C1–C3: Cluster 1–3.

In total, 37 amino acids and their derivatives were significantly changed (1.5 times fold, *p* < 0.05), which could be categorized into three clusters. The relative content of compounds (*O*-Acetylserine, 3,4-Dihydroxy-*DL*-phenylalanine, γ-Glu-Cys, Asp-phe, and *D*-Alanyl-*D*-Alanine) in Cluster 1 significantly reduced after 7 h of HRH treatment (Figure 3, Cluster 1). Both short and long time HRH treatment significantly decreased the relative concentration of compounds in Cluster 2 (Figure 3, Cluster 2). In particular, glutamic acid and aspartic acid were in this cluster. Most of the changed amino acids and derivatives were in Cluster 3; the relative concentration of them significantly increased after HRH treatment, especially for a long time, including six essential amino acids and three non-essential amino acids. GABA was in this cluster (Figure 3, Cluster 3). The relative concentration of six essential amino acids, methionine, lysine, leucine, isoleucine, threonine, and valine, increased 11.62, 6.42, 3.41, 3.18, 2.78, and 2.30 times after HRH (7 h) treatment, respectively. The relative concentration of three non-essential amino acids, glutamine, tyrosine, and histidine, increased 5.98, 3.95, and 3.76 times after HRH (7 h) treatment, respectively.

#### 3.3.3. The Change of Individual Phenolic Acids during GABA-Enrichment Processing

**Figure 4:**
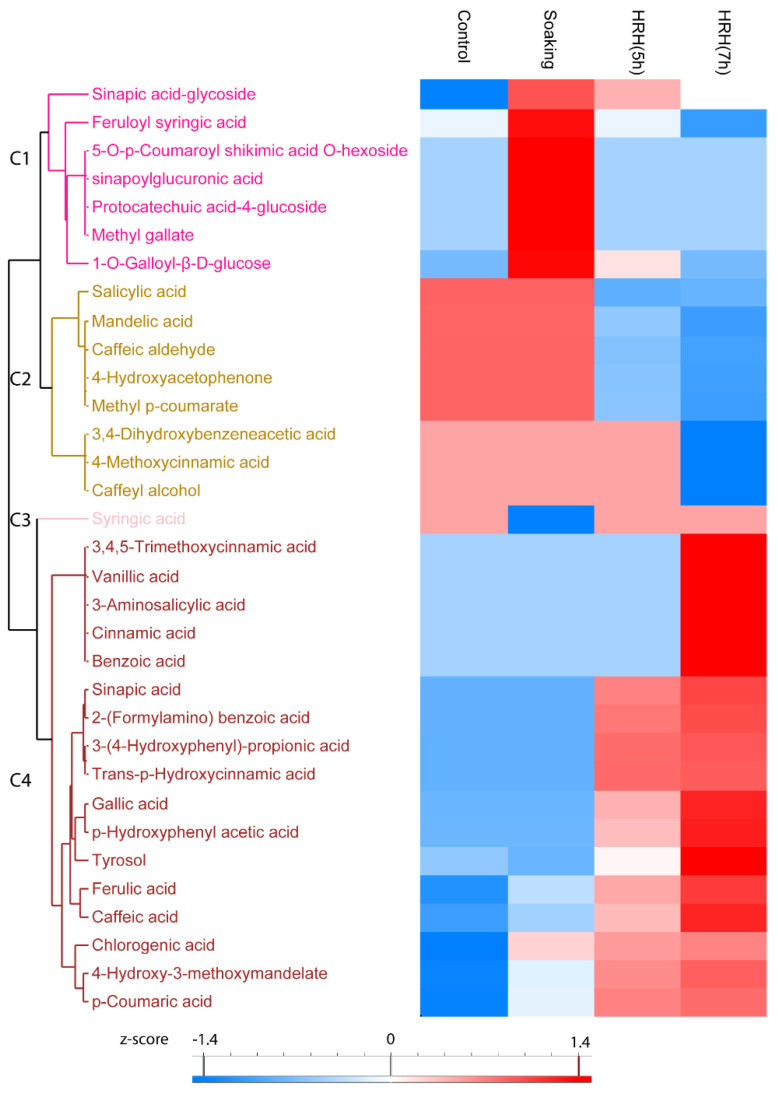
Hierarchical cluster analysis based on peak area of phenolic acids. C1–C4: Cluster 1–4.

In total, the relative content of 33 phenolic acids significantly changed (1.5 times fold change, *p* < 0.05) during processing, which can be divided into four clusters. The relative concentration of sinapic acid-glycoside, feruloyl syringic acid, 5-*O*-*p*-Coumaroyl shikimic acid *O*-hexoside, sinapoylglucuronic acid, protocatechuic acid-4-glucoside, methyl gallate, and 1-*O*-Galloyl-β-*D*-glucose in Cluster 1 significantly increased after soaking and decreased after HRH treatment (Figure 4, Cluster 1). Moreover, the relative concentration of eight phenolic acids, including salicylic acid, mandelic acid, etc., in Cluster 2 was higher in the control and soaked group but lower in the HRH-treated group, especially for a long time (Figure 4, Cluster 2). The relative content of syringic acid decreased only after soaking (Figure 4, Cluster 3). Moreover, the most frequently reported phenolic acids in mung beans, including ferulic acid, caffeic acid, gallic acid, and coumaric acid, accumulated during HRH treatment and reached the highest level after 7 h. (Figure 3, C4).

#### 3.3.4. The Changes of Individual Organic Acids during GABA Enrichment Processing

**Figure 5:**
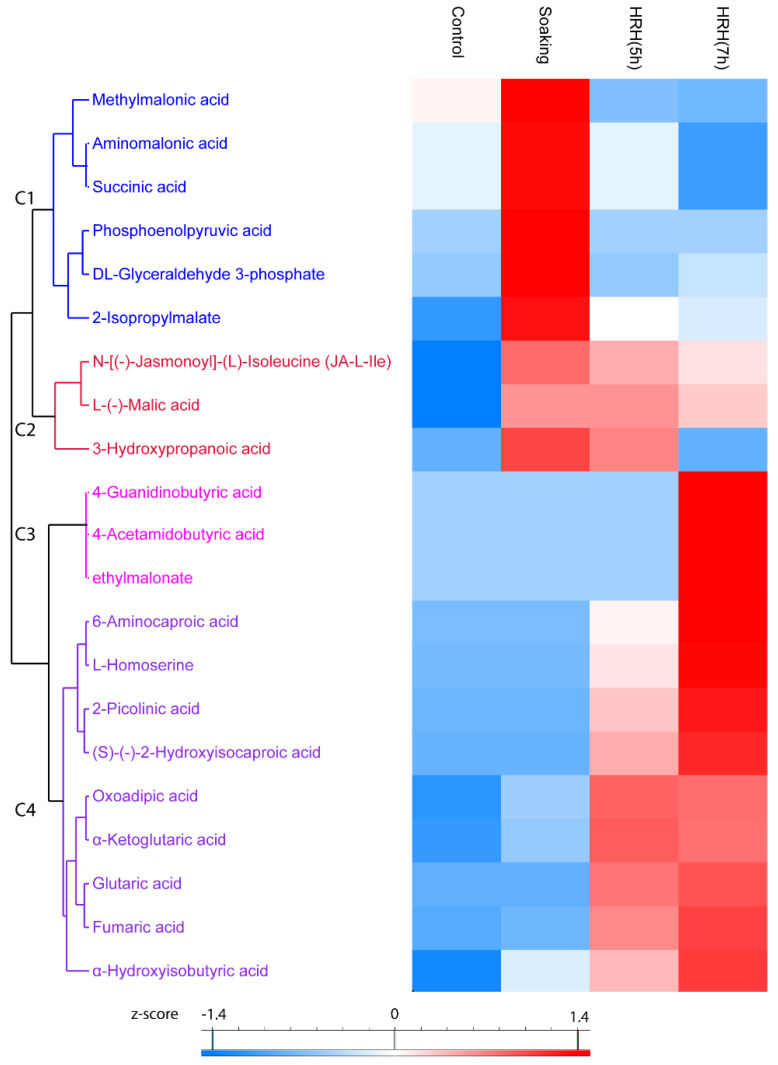
Hierarchical cluster analysis based on peak area of organic acids. C1–C4: Cluster 1–4.

In total, 21 organic acids significantly changed (1.5 times fold, *p* < 0.05) during processing, which can be divided into four clusters. In Cluster 1, the relative concentration of these compounds was the highest after soaking and decreased during HRH treatment. For example, the relative concentration of methylmalonic acid, aminomalonic acid, and succinic acid was significantly lower than that of the control group after a long time HRH treatment (Figure 5, Cluster 1). In Cluster 2, the relative amount of these organic acids (*N*-[(-)-Jasmonoyl]-(*L*)-Isoleucine (JA-*L*-Ile), *L*-(-)-Malic acid, and 3-Hydroxypropanoic acid) was higher after soaking and slightly decreased during HRH treatment, but still higher than that of the control group, except for 3-hydroxypropnoic acid (Figure 5, Cluster 2). In Cluster 3, the relative concentration of three organic acids (4-guanidinobutyric acid, 4-acetamidobutyric acid, and ethylmalonate) only significantly increased after a long time of HRH treatment (Figure 5, Cluster 3). In Cluster 4, the relative concentration of these organic acids was higher after HRH treatment. Of note, the relative content of oxoadipic acid and α-ketoglutaric acid was higher in HRH (5 h) than in HRH (7 h). However, for the other seven organic acids in this cluster, the relative concentration of them was higher after HRH (7 h) treatment (Figure 5, C4). In addition, the relative concentration of fumaric acids was more than 15 and 18 times higher after HRH (5 h) and HRH (7 h) treatment than that of the control group, respectively. Moreover, the abundance of α-ketoglutaric acid increased more than 22 and 21 times after HRH (5 h) and HRH (7 h), respectively.

## 4. Discussion

In the present study, 496 primary and secondary metabolites in mung beans, were identified and relatively quantified during GABA-enrichment processing by using widely targeted metabolomics techniques. Suitable processing steps can be selected based on desired bioactive compounds and expected bioactivities. The changes of bioactive molecules during processing provide us clues on the additional nutritional value of GABA enriched mung beans.

In the present study, the GABA content of mung beans was accumulated during HRH treatment. This could be ascribed to the high temperature and low-oxygen environment during HRH processing. This condition was stressful for mung beans [20]. GABA was probably accumulated through the GABA shunt to overcome this stress condition [25]. In consistent with this notion, the amount of glutamic acid, which is the precursor of GABA shunt, significantly decreased during HRH treatment. While the amount of the other nine free amino acids increased, this could be due to the degradation of proteins during stress conditions to fulfill the metabolic demands of mung beans [26].

Mung bean is well known for its high level of flavonoids. Flavonoids are the most important secondary metabolites and contribute to the antioxidative activities of mung beans [3,21] They are also related to the hypoglycemic, hypolipidemic, anti-cancer, immunomodulatory activity, and anti-melanogenesis properties of mung beans [4]. The total relative content of flavonoids increased after the GABA enrichment processing. The highest level of flavonoids was observed after soaking and slightly decreased after HRH treatment. Thus, soaking was a critical step for the enrichment of total flavonoids. However, the changes of individual flavonoids were different. In detail, the glycosylated flavonoids were enriched during soaking and decreased to a similar level to the control group after HRH treatment (Figure 2, Cluster 1). While, un-glycosylated flavonoids were significantly upregulated after HRH treatment (Figure 2, Cluster 3). In contrast to mung beans, the glycosylation was decreased in soyabeans during soaking because of the increased β-glucosidase activity [27]. Thus, the general knowledge of soyabeans on flavonoids’ glycosylation cannot be generalized to mung beans. The reason and effect of diverse changes of individual flavonoids in mung beans during processing need to be further investigated.

Currently, the precise biological functions of flavonoids’ glycosylation are still under debate. On the one hand, O-glycosylation was shown to generally reduce the diverse bioactivities of flavonoids, including antioxidant, antidiabetics, anti-inflammation, antibacterial, antifungal, and antitumor activity, etc. [28]. On the other hand, O-glycosylation enhanced anti-HIV activity, tyrosinase inhibition, anti-rotavirus activity, anti-stress activity, etc., of flavonoids [28]. The precise function of flavonoids’ glycosylation in mung beans needs further investigation.

Phenolic acids also contribute significantly to the antioxidative activity of mung beans [29]. The relative content of phenolic acids was highest after soaking and decreased during HRH treatment. However, it was still higher than that of the control. The relative content of glycosylated phenolic acids was highest after soaking. It was shown that the glycosylated phenolic acids were the storage of precursors of more bioactive compounds. However, they have higher bioavailability than their aglycones in vivo [30]. The most frequently reported phenolic acids, including ferulic acid, caffeic acid, gallic acid, and coumaric acid, significantly increased around 2–4 times during HRH treatment. Thus, HRH treatment increased not only the level of GABA but also the above three abundant phenolic acids.

Furthermore, three compounds in the TCA cycle changed significantly during GABA-enrichment processing. For example, the amount of α-ketoglutaric acid increased more than 20 times after HRH treatment. α-ketoglutarate is a key molecule in the Kreb cycle contributing to the overall rate of the citric acid cycle [31]. Physiological functions of α-ketoglutaric acid are various, such as modulating protein synthesis, bone development, and maintaining homeostasis of the immune system [31]. In recent studies, α-ketoglutarate was found to extend the lifespan of aging mice and adult *Caenorhabditis elegans* [32,33]. Our results implied that the short time HRH treatment was sufficient to accumulate α-ketoglutarate in mung beans.

Seven hours compared to 5 h HRH treatment only led to a marginal increase in GABA content. In addition, the increasement of α-ketoglutaric acid, another important bioactive molecule, was higher in 5 h than that in 7 h HRH treatment. Seven hours of HRH treatment also decreased the total relative content of flavonoids and phenolic acids. Thus, in our opinion, soaking together with 5 h HRH treatment might be suggested for producing GABA enriched bio-functional mung bean food, which also contained higher content of α-ketoglutaric acid and polyphenols.

HRH treatment required less water and time comparing to germination and fermentation. Thus, it is a promising technique for improving mung bean’s functional components and physicochemical properties [34,35]. Fukumori et al., applied this technique on GABA enrichment in rice, wheat, soyabean, adzuki bean, etc. [20]. Based on Fukumori’s and our results, HRH technique is a useful tool that can be applied to various seeds and other materials to enrich biomolecules.

## 5. Conclusions

HRH treatment was a sufficient approach to enrich GABA and other bioactive molecules, including flavonoids, phenolic acids, and organic acids in mung beans. It might be applied in other materials as well. Depending on desired bioactive compounds, soaking, 5 h or 7 h HRH treatment could be selected. Our results deepened the understanding on the change of bioactive compounds in mung beans during GABA enrichment processing.

## Figures and Tables

**Table 1:** Changes of different types of metabolites during GABA-enrichment processing, peak area of each metabolite was used (×105). The content of γ-aminobutyric acid was displayed in mg/100 g.

	Control	Soaking	HRH ^1^ (5 h)	HRH (7 h)
**Total Flavonoids**	**1049.61 ± 13.24 ^d^**	**1553.21 ± 30.51 ^a^**	**1346.67 ± 5.77 ^b^**	**1250 ± 51.96 ^c^**
Flavonols	410.76 ± 13.36 ^c^	652.61 ± 35.39 ^a^	481.89 ± 11.75 ^b^	370.15 ± 5.27 ^d^
Flavones	353.54 ± 13.28 ^c^	539.57 ± 13.88 ^a^	503.7 ± 1.82 ^b^	503.16 ± 30.4 ^b^
Flavanones	133.41 ± 6.55 ^b^	176.47 ± 3.16 ^a^	173.73 ± 4.41 ^a^	197.25 ± 17.41 ^a^
Isoflavones	122.54 ± 12.01 ^b^	157 ± 6.57 ^a^	155.17 ± 6.57 ^a,b^	148.95 ± 1.03 ^a,b^
Flavanols	25.75 ± 5.84	22.30 ± 0.90	28.55 ± 4.92	27.46 ± 0.64
Chalcones	1.93 ± 0.20	2.34 ± 0.10	2.38 ± 0.09	2.20 ± 0.18
Proanthocyanidin	1.68 ± 0.20	2.69 ± 0.27	2.72 ± 0.15	2.93 ± 0.22
**Total Amino acids and derivatives**	**1783.34 ± 13.29 ^c^**	**1886.67 ± 15.28 ^b^**	**1870 ± 34.64 ^b^**	**2170.00 ± 79.37 ^a^**
Essential Amino acids	614.96 ± 26.43 ^c^	696.03 ± 3.13 ^b^	740.21 ± 29.99 ^b^	880.63 ± 44.27 ^a^
Nonessential Amino acids	815.20 ± 41.42 ^a^	791.70 ± 39.46 ^a^	697.71 ± 14.46 ^b^	813.10 ± 35.17 ^a^
Amino acids derivatives	353.19 ± 8.15 ^c^	396.68 ± 28.58 ^b,c^	433.72 ± 8.30 ^a,b^	474.65 ± 14.92 ^a^
**Total Phenolic acid**	**532.94 ± 22.19 ^c^**	**620.67 ± 24.12 ^a^**	**584.23 ± 2.26 ^a,b^**	**566.29 ± 3.11 ^b^**
**Total Organic Acid**	**1146.64 ± 25.52 ^b^**	**1545.90 ± 79.79 ^a^**	**1310.12 ± 46.50 ^b^**	**1281.03 ± 43.17 ^b^**
**Total Nucleotides and derivatives**	**631.87 ± 221.95**	**1130.79 ± 156.50**	**758.52 ± 217.59**	**1145.82 ± 408.03**
**Total lipids**	**1590.74 ± 22.47 ^b^**	**1289.36 ± 63.20 ^c^**	**1763.31 ± 93.24 ^a^**	**1868.51 ± 188.16 ^a^**
Free fatty acids	947.92 ± 7.15 ^b,c^	822.06 ± 49.47 ^c^	1077.33 ± 59.54 ^b^	1304.97 ± 120.83 ^a^
Phospholipids	624.59 ± 27.58 ^b,c^	450.23 ± 21.66 ^a^	661.94 ± 51.77 ^a,c^	538.86 ± 66.33 ^a^
Glycerides	18.23 ± 1.55	17.06 ± 0.67	24.03 ± 4.13	24.68 ± 1.56
**Total Saccharides, Alchols and vitamins**	**757.12 ± 11.92 ^c^**	**746.48 ± 48.25 ^c^**	**926.25 ± 35.59 ^b^**	**1076.67 ± 37.86 ^a^**
Saccharides and Alcohols	481.72 ± 12.75 ^a^	490.93 ± 36.30 ^a^	571.44 ± 33.77 ^b^	701.61 ± 28.64 ^a^
Others	224.65 ± 22.96 ^b^	203.65 ± 12.40 ^b^	301.36 ± 56.15 ^a^	319.19 ± 8.52 ^a^
Vitamins	50.75 ± 2.57	51.89 ± 1.52	53.46 ± 1.38	53.71 ± 3.10
**Alkaloids**	**466.87 ± 6.46 ^b^**	**479.89 ± 4.08 ^a,b^**	**470.34 ± 4.97 ^a,b^**	**483.97 ± 6.87 ^a^**
**Terpenoids (Saponins)**	**246.84 ± 9.66 ^b^**	**314.68 ± 10.98 ^a^**	**234.43 ± 15.03 ^b^**	**172.52 ± 6.71 ^c^**
**Coumarins**	**129.97 ± 4.79 ^b^**	**157.76 ± 14.01 ^a^**	**146.50 ± 7.81 ^a^**	**145.65 ± 4.19 ^a^**
**Lignans**	**2.75 ± 0.11**	**3.15 ± 0.06**	**2.93 ± 0.07**	**2.76 ± 0.19**
**Cholestrol**	**0.75 ± 0.09**	**0.60 ± 0.33**	**0.76 ± 0.08**	**0.70 ± 0.08**
**γ-aminobutyric acid**	**2.86 ± 0.17 ^d^**	**9.37 ± 0.43 ^c^**	**112.82 ± 2.68 ^b^**	**120.37 ± 3.08 ^a^**

^1^ HRH: heat and humidity treatment; ^a,b,c,d^: *p* < 0.05.

## Data Availability

The data presented in this study are available in article and supplementary material.

## Supplementary Materials

The following supporting information can be downloaded at: https://www.mdpi.com/article/10.3390/foods11101423/s1, Table S1: identified and quantified molecules during GABA-enrichment processing. HRH: heat and humidity treatment.
